# Synchrony and multimodality in the timing of Atlantic salmon smolt migration in two Norwegian fjords

**DOI:** 10.1038/s41598-021-85941-9

**Published:** 2021-03-22

**Authors:** Helge B. Bjerck, Henning A. Urke, Thrond O. Haugen, Jo Arve Alfredsen, John Birger Ulvund, Torstein Kristensen

**Affiliations:** 1 Faculty of Biosciences & Aquaculture, Nord University, Bodø, Norway; 2 INAQ AS, Trondheim, Norway; 3 Faculty of Environmental Sciences & Natural Resource Management, Norwegian University of Life Sciences (NMBU), Ås, Norway; 4 Faculty of Information Technology & Electrical Engineering, Norwegian University of Science & Technology (NTNU), Trondheim, Norway

**Keywords:** Animal migration, Behavioural ecology, Freshwater ecology, Ecology

## Abstract

The timing of the smolt migration of Atlantic salmon (*Salmo salar*) is a phenological trait increasingly important to the fitness of this species. Understanding when and how smolts migrate to the sea is crucial to understanding how salmon populations will be affected by both climate change and the elevated salmon lice concentrations produced by salmon farms. Here, acoustic telemetry was used to monitor the fjord migration of wild post-smolts from four rivers across two fjord systems in western Norway. Smolts began their migration throughout the month of May in all populations. Within-population, the timing of migration was multimodal with peaks in migration determined by the timing of spring floods. As a result, migrations were synchronized across populations with similar hydrology. There was little indication that the timing of migration had an impact on survival from the river mouth to the outer fjord. However, populations with longer fjord migrations experienced lower survival rates and had higher variance in fjord residency times. Explicit consideration of the multimodality inherent to the timing of smolt migration in these populations may help predict when smolts are in the fjord, as these modes seem predictable from available environmental data.

## Introduction

Juvenile Atlantic salmon (*Salmo salar*), known as smolts, migrate to the sea from their natal rivers as a part of their natural life cycle. Throughout the range of this species, this migration occurs mostly during spring and early summer when smolts are 1–6 years old and at a size of 12–25 cm^1,2^. However, the timing of migration within each population is highly variable, both within and between years^3,4^. In addition, climate change and the rise of aquaculture are significantly altering the fitness consequences of this variation which may have profound consequences for the overall health of these populations. As a result, understanding between- and within-population variation in migration timing, and the factors that structure this variation, has become more important than ever.

Return rates to the river indicate that an optimal migration time exists^5^. There seems to be a consistent positive effect of high sea surface temperatures during outmigration on return rates across Norwegian populations, likely due to increased prey availability^6–8^. However, smolts arriving in the sea too late may run the risk of missing out on optimally sized prey items^9^. As such, smolts face the fundamental problem of needing to determine the optimal time to migrate using conditions within freshwater when the conditions that determine this optimality are in the ocean, far away in space and time. As there is no guarantee that the freshwater environment and the marine environment will change in tandem, this potential for phenological match-mismatch will likely grow as the climate changes^1,10,11^.

Smolts migrating from the river to the open sea generally experience substantial mortality rates, primarily because of predation, but also because of the physiological and metabolic challenge of migrating long distances to drastically different environments^12,13^. Adding to this, the post-smolt migration period is the period in the salmon lifecycle where it is the most susceptible to salmon lice (*Lepeophtheirus salmonis*) infestation^13^. Net-pen production of salmonids has vastly increased the number of available hosts for parasitic salmon lice, consequently increasing the numbers of these ectoparasites in the waters surrounding fish farms^14,15^. As most fish farms are located in the coastal archipelago between the mouth of the fjord and the open sea, post smolts must necessarily risk infestation to complete their migration. However, modelling and monitoring of salmon lice abundance has shown that the risk of infestation for a migrating smolt can vary significantly through time and space, as the density of fish farms can differ widely among regions and migration routes while salmon lice abundance increases substantially with increasing temperatures through spring^14,16^. As a result, models that estimate salmon lice induced mortality are especially sensitive to deviations from assumptions about when smolts begin their migration and how quickly they reach the open sea^16,17^. As such, there is a need for a better understanding of these phenological traits.

Previous research has clearly shown that smolts use environmental cues to decide when to begin their migration^12^. Photoperiod is well-established as a zeitgeber for the parr-smolt transformation^18–21^, the physiological transformation preparing the fish for seawater entry, and also as a proximal trigger of migration^22,23^. Other environmental triggers that have been shown to be important are river temperature and river discharge^24,25^. However, river conditions in rivers vary widely throughout the species range. For example, in many salmon rivers, increases in air temperature through spring will have the effect of warming the river. However, many Norwegian rivers are primarily fed by snowmelt throughout the migration period, such that increases in air temperature often lead to an influx of cold water to the river. Additionally, there may be constant daylight throughout the migration period at high latitudes, such that photoperiod would be a poor cue (e.g.^26^). As a result, smolts in different rivers must weigh these environmental factors differently when preparing to migrate. Indeed, common-garden studies have documented between-population variation in migration timing, suggesting that some of this variation is genetically controlled^27–30^.

Here, we used acoustic telemetry to monitor the fjord migration of salmon smolts from four rivers located within two fjords in western Norway. This was coupled with environmental data from each river to investigate the proximal cues for migration timing. Then, mark-recapture models were used to estimate survival through the river-fjord-coast migration route.

Acoustic telemetry has become a widely used method to monitor the movements of aquatic animals^31,32^ and it has turned out to be especially useful for monitoring anadromous salmonids (e.g.^33–35^). Pre-smolts can be captured and tagged weeks before their migration begins such that the stress of tagging has likely abated when the behavior is observed. Further, this method allows us to observe migration timing in a manner that is insensitive to changes in the turbidity and water level of the river. Finally, the placement of receivers within the river and throughout the fjord can enable sequential registrations of the same smolt as it migrates through the coastal area.

Here, we addressed the following research questions. First, what proximal cues trigger smolts to begin migration and how do these cues structure within-population variance in migration timing? Then, when do smolts from populations within long fjords begin their migration, and how long until they enter the coastal area? Lastly, how do survival rates in the fjord differ through space and time?

## Methods

### Study system

**Figure 1:**
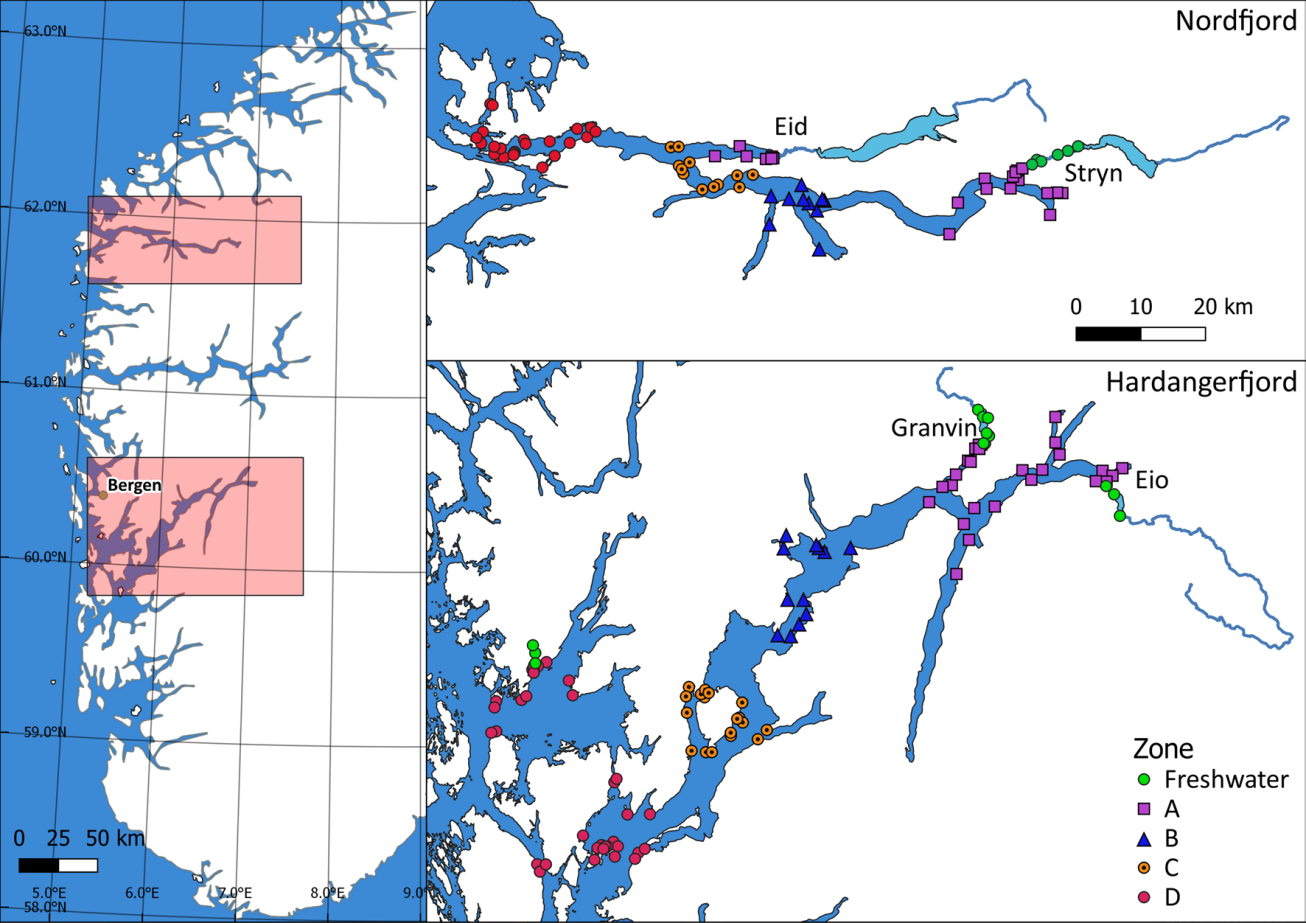
Map of study system showing locations of acoustic receivers along with the zone classification of these receivers. Figure produced using QGIS v3.10.2, qgis.org.

The study was conducted in four rivers across two fjords, Hardangerfjord and Nordfjord, in western Norway (Fig. 1). These rivers are considered nationally/regionally important for maintaining viable populations of Atlantic salmon and are located in areas impacted by aquaculture. All four rivers drain into long fjords, and their watersheds are primarily mountainous. The rivers range in length from 2 to 10 km, and each flows out of a large lake.

Hardangerfjord has a total length of approximately 160 km, making this fjord the second longest in Norway. This is a complex fjord, varying in width from 2 to 7 km, with many sidearms, inlets, and islands. Two rivers, Granvin and Eio, with outlets deep within this fjord were sampled for this study.

Granvin empties into Granvinsfjorden, an arm of inner Hardangerfjord. The watershed encompasses 177 km^2^ and the 25th percentile of elevation of this watershed is 461 m above sea level. The studied portion of this river is 2.4 km long and runs between a 4.1 km^2^ lake and the outlet into the fjord. Here, the estimated annual Atlantic salmon smolt production is 6779 smolts^36^. This river is protected from hydropower expansion.

The river Eio drains into Eidfjord, the innermost arm of Hardangerfjord. This river has had a hydropower station since the mid-70s leading to a significant diversion of water away from Eio, reducing the watershed from 1015 to 640 km^2^. The 25th percentile of elevation of this watershed is 1108 m above sea level. This river is the shortest of the sampled rivers at a length of 2 km running between a 3.7 km^2^ lake and the river mouth. The annual Atlantic salmon smolt production is estimated to be 15,479 smolts^36^.

Nordfjord is the sixth longest fjord in Norway, spanning 106 km. Much of Nordfjord has been designated as a National Salmon Fjord by the Norwegian Environment Agency and is protected from aquaculture expansion. Additionally, both sampled rivers in this fjord, Eid and Stryn, have been designated as National Salmon Rivers. This designation provides for more stringent regulations to ensure the health and diversity of these salmon populations.

The river Eid runs from Hornindalsvatnet (52 m above sea level, 50.4 km^2^), Europe’s deepest lake, and has its outlet in Eidsfjord, an arm of Nordfjord. The river is approximately 10 km long and the total river area is ca. 0.35 km^2^. The watershed encompasses 422 km^2^ and the 25th percentile of elevation is 213 m above sea level. The river is somewhat regulated by hydropower as 20 km^2^ of the watershed above Hornindalsvatnet has been diverted, but the watershed has been protected from further hydropower development since 1973. The estimated annual smolt production is 33,191 smolts^36^.

The river Stryn is a 10 km long stretch of river between a 22.9 km^2^ lake and inner Nordfjord. The estimated annual smolt production is 48,501 smolts^36^. The watershed spans 537 km^2^ and the 25th percentile of elevation is 627 m. This watershed is a pristine watershed with no hydropower development.

### Fish sampling

Salmon pre-smolts were captured by electrofishing during April. Fish were kept in 400 L covered tanks with running river water overnight. Individual fish were transferred into an anaesthetic bath (60 mg L^−l^ tricaine methanesulphonate (MS-222)). After reaching full anaesthesia, all individuals were measured to the nearest mm (total length), weighed to the nearest 0.1 g and placed in a v-shaped surgical trough with a continuous anaesthetic flow over the gills for the entire procedure (40 mg L^−l^ MS-222). A small fin clip of the pelvic fin was taken for DNA analyses. Acoustic tags were sterilized in ethanol, allowed to air dry, and inserted intraperitoneally through a small incision (~ 10 mm) slightly offset of the ventral line, about 2 cm behind the pectoral fin. The incision was closed with three interrupted double surgical knots using non-absorbent 4-0 monofilament suture and sealed with a tissue adhesive (monomeric n-butyl-2-cyanoacrylate).

After the surgical procedure, the tagged smolts were kept in 60 L recovery tanks continuously refreshed with water until they reached full consciousness (1–2 min). The smolts were subsequently released near their respective catch sites within four hours after tagging.

The surgical protocol for implanting acoustic tags followed the general recommendations given by Mulcahy^37^ and Cooke and Wagner^38^*.* The experimental protocol was approved by the Norwegian Animal Research Authority (ID 15471 and ID 12002) and the provincial government as per the Norwegian Regulation on Animal Experimentation 18.06.2015, § 6 and 12. The tagging permits specified a minimum tagging size of 12.5 cm. However, due to a communication error, 85 out of the 377 individuals tagged were below this threshold. We were not able to detect any difference in the performance of these individuals and those that were above the threshold (see “Discussion”). All personnel involved in the tagging and handling of fish were trained and familiar with FELASA guidelines, and the study was planned and conducted in accordance with ARRIVE guidelines^39^.

Two different types of acoustic tags (ID-LP7 and D-LP7) were used, both manufactured by Thelma Biotel AS, Trondheim, Norway. Both transmit unique ID codes every 30–90 s and have a battery life of roughly 150 days, but D-LP7 also transmits depth data with a resolution of 0.2 m. ID-LP7 transmitted on the protocol R64K, now known as A69-1303, while D-LP7 transmitted on the protocols S64K and S256 in Hardangerfjord and Nordfjord, respectively. The transmitting range of these tags is highly dependent on the acoustic conditions in the environment and can vary from 100 to 400 m. The tags that only transmitted ID were 7.3 mm in diameter and 17 mm long, with a weight in air of 1.8 g. The tags that also transmitted depth data were slightly longer with a length of 21.5 mm and a weight in air of 2.0 g. The mean tag burden across all tagged fish was 9.8% of body weight, with a standard deviation of 2.2%.

In Nordfjord, 118 (134 mm ± 11.6; length ± SD) pre-smolts were tagged in the river Stryn in 2017. In 2018, sampling was extended to include the river Eid, such that 34 (129 mm ± 11.1) and 66 (131 mm ± 10.2) pre-smolts were tagged in the rivers Stryn and Eid, respectively. In Hardanger, 74 (137 mm ± 10.9) and 86 (141 ± 15.4) pre-smolts were tagged in Eio and Granvin, respectively (Table 1).

**Table 1:** Sample sizes and quantiles of fjord entry dates and arrival dates in the outer fjord for each river-year.

River-year	# Tagged	# Migrated	Proportion migrated	Quantiles of fjord entry dates			# of Migrants detected in zone D	Proportion of migrants detected in zone D	Quantiles of arrival to outer fjord		
				25%	50%	75%			25%	50%	75%
Granvin 2018	86	66	0.77	2018-05-06	2018-05-12	2018-05-24	25	0.38	2018-05-17	2018-05-22	2018-05-27
Eio 2018	74	57	0.77	2018-05-10	2018-05-17	2018-05-30	13	0.23	2018-05-22	2018-05-22	2018-06-01
Eid 2018	66	59	0.89	2018-05-06	2018-05-10	2018-05-25	31	0.53	2018-05-08	2018-05-12	2018-05-14
Stryn 2018	33	19	0.58	2018-05-08	2018-05-16	2018-05-29	6	0.32	2018-05-21	2018-06-06	2018-06-15
Stryn 2017	118	71	0.60	2017-05-06	2017-05-16	2017-05-17	27	0.38	2017-05-12	2017-05-16	2017-05-23
Total:	3778	272	0.72				102	0.38			

### Receiver network

In Nordfjord, 71 Innovasea (VR2W) receivers were deployed throughout the fjord and within the rivers Stryn and Eid. The majority of these were deployed in April of 2017, but the receiver network was expanded to include Eidsfjord and the river Eid in April of 2018.

In Hardanger, 106 Thelma Biotel (TBR 700) receivers were placed throughout the fjord and within the rivers Granvin and Eio. In addition to receiving signals from acoustic tags, these receivers also collected data on noise levels and water temperature, logging a summary of these variables for each 10 minute interval. Using the data on background noise levels, a signal-to-noise ratio (SNR) was calculated for each putative detection.

All receivers were moored in place in a downward orientation and kept roughly at 4 m from the surface by buoys. Receivers were maintained on a bimonthly basis to ensure continuous operation.

### Quality control

Each individual’s detection trajectory was individually inspected to identify possible false detections. Detections were removed if they were at an unlikely position relative to other detections (e.g. detected in the outer fjord before initiation of migration). Mortalities can generally be identified when smolts die within detection range of our receivers, if this occurs in saltwater, as the smolts will be stationary or appear to be going back and forth between neighboring receivers. Depth data, when available, can also provide an indication of possible mortalities or tag loss.

However, it is difficult to ascertain the fate of individuals that do not enter the sea. These individuals could represent river mortalities, tag losses, or simply individuals that have chosen not to migrate this year. As a result, these fish are not included in any analyses.

### Migration metrics

Fjord entry date is defined as the date and time of the first detection at a receiver in the marine environment after the last detection at a receiver in freshwater. As all the rivers in this study are relatively short, the amount of time between beginning downstream migration and entering the sea should be negligible. Stryn is the longest of the rivers and smolts here could undertake the 10 km journey from the release point to the river mouth in under four hours. However, sparse and ambiguous data within the river made it difficult to estimate progression rates within the river for all fish. Previous research in a similar river showed that the average within-river migration speed for wild smolts was 45 km/day^25^, indicating that migrating through the river should take less than 1 day in all of the rivers studied here. As a result, fjord entry date served as a standardized measure of when the migration began across rivers.

The distributions of individual fjord entry dates were clearly multimodal within each river, indicating that these were mixture distributions. Gaussian clustering was therefore used as an objective method to identify how many modes there were and when they occurred. This clustering was performed with the function stepFlexmix in the R package flexmix^40,41^. For each permutation of river and year, (i.e. river-year), five candidate models were fitted, fitting one to five normal distributions to the distributions of fjord entry dates, with 500 replicates of each model. The most supported candidate model for each river-year was chosen by minimizing the Bayesian Information Criterion (BIC).

The resulting clusters were classified as being narrow distributions if their standard deviation was less than 4.5 days. Narrow distributions indicate that many fish are migrating at roughly the same time, such that they are likely triggered by the same coinciding environmental cue, whereas broad distributions could indicate migration that is not motivated by a common cue. Additionally, the results of these analyses provide posterior probabilities that indicate whether an individual can be assigned to a cluster. The maximum posterior probability that an individual belonged to a narrow cluster was used as a metric indicating that a fish migrated simultaneously as others in the population.

### Environmental correlates of migration

Temperature was recorded every 10 minutes by each acoustic receiver in Hardanger. Receivers deployed in these rivers could be used to get near real-time temperature data. For the rivers in Nordfjord, temperature was recorded using Innovasea Minilog II temperature loggers deployed in the river.

Water discharge data for the rivers Eio, Eid, and Stryn came from the Norwegian Water Resources and Energy Directorate (NVE). This agency operates discharge monitoring stations in most major Norwegian rivers based on stage-discharge relationships. However, NVE does not maintain a station in Granvin. Here we installed a pressure sensor at the study site and converted stage to discharge using a relationship developed through hydraulic modelling.

Generalized linear mixed modeling was used to model the migration probability across rivers given the river conditions on each day^42^. This was done using the glmer function in the R package lme4^43^. The response was modelled as a binomial distribution given the number of individuals that migrated and the number of individuals that could have migrated on a given day within a given river year. Models were built with forward selection using AIC as the criterion. River-year was used as a random effect throughout. Additional terms in the model were only included if the change in AIC was greater than 2. Given the magnitude of variation in discharge between rivers, both discharge and day-to-day change in discharge were scaled and centered within-river (mean = 0; SD = 1).

### Survival analysis

Survival was estimated using mark-recapture analysis under a Cormack-Jolly-Seber (CJS) model structure^44^ using the R package RMark^45^. This program allows an R interface with the program MARK^46^ which is designed for estimating survival using mark-recapture methods. These models use a temporal capture history for estimating probability of survival and probability of detection simultaneously between and within each discrete sampling event. Given that outmigrating smolts can be viewed as moving directionally in one dimension, we can use a space-for-time substitution framework to estimate sequential survival between groups of receivers in the fjord and the detection probability at each of these groups^47,48^.

To aid this process, the receivers in each fjord were grouped into discrete zones (Fig. 1). In both fjords, zone A represents the fjord arms wherein the mouth of each river is located. Zones B and C represent central sections of the fjord. Zone D represents the outer fjord, which opens into the coastal archipelago, and, eventually, the open sea. Smolts originating from the river Eid have a substantially shorter fjord migration, and therefore do not move through any zone B. As such, models that allowed survival and detection rates to vary between zones were fitted.

Models were run independently for the two Hardanger populations (Granvin and Eio), Eid, and both years of data from Stryn. The effect of year was tested in the model for both years of data from Stryn and the effect of river of origin was tested in the model for Hardanger data. The effects of smolt length, fjord entry date, the position of the sun with respect to the horizon at fjord entry (see Supplementary Materials), and the maximum posterior likelihood of belonging to a narrow distribution were tested as continuous covariates in all three datasets. Models were built using forward selection and the most parsimonious model with a change in AIC of less than two from the model with lowest AIC value was selected as the most supported model by the data. Relatively low sample sizes and the large number of parameters that each CJS model needs to estimate precluded our ability to test complex models. Therefore, interactions between effects were not explored and parsimonious models were favored, as the addition of further parameters often led to problems with convergence.

Probability of detection in the outermost fjord zone was fixed to 1 during model selection as we cannot separate the probability of survival and probability of detection in the final zone. The estimate for probability of survival given here represents the minimum possible probability of survival. For the visualization of the results, we also ran a model with the final probability of detection set to a more realistic value of 0.65 in order to acquire estimates of the expected survival. This value is similar to the estimated detection probability of other zones in the fjord with similar receiver density (Supplementary Table S3).

As we only use smolts that successfully migrated from the river in these models, the probability of survival from the river to zone A was fixed to 1 in order to reduce the number of parameters the models needed to estimate.

## Results

Overall, 72% of tagged individuals were subsequently detected as migrators (Table 1). Three of these individuals were removed from all analyses, as the true fate of these individuals was difficult to ascertain due to the number of unreliable detections.

### Fjord entry date

**Figure 2:**
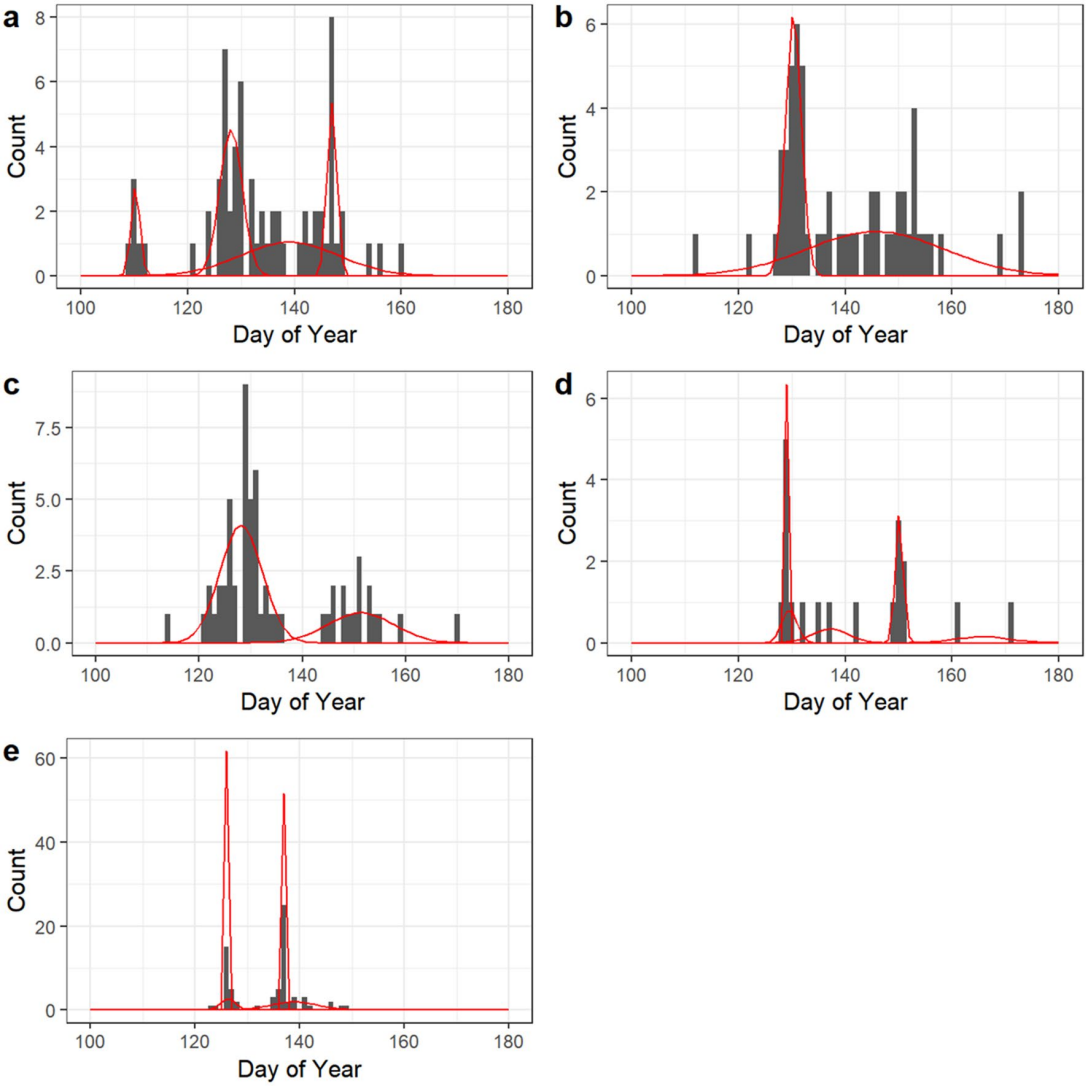
Histograms of fjord entry dates for each river-year, along with results of Gaussian clustering of these fjord entry dates. Plots represent (**a**) Granvin 2018, (**b**) Eio 2018 , (**c**) Eid 2018, (**d**) Stryn 2018, and (**e**) Stryn 2017. Clusters are scaled according to the number of individuals assigned to that cluster.

Median and 25% quantiles of fjord entry dates were largely similar between rivers and years, differing up to 6 days (Table 1). However, quantiles belie the shapes of these distributions. Smolts from all four rivers displayed multimodality in the distribution of fjord entry dates, and these modes largely occurred at the same time across rivers (Fig. 2).

Gaussian clustering of fjord entry dates revealed that two clusters were most supported by the BIC selection procedure for both Eio and Eid fjord entry dates, while four clusters were most supported for Granvin fjord entry dates. For the data from Stryn, four and five clusters were most supported by the data from 2017 and 2018, respectively (Fig. 2, see also Supplementary Figure S1).

Both Eio and Eid displayed one initial narrow cluster, with a broader cluster afterwards. Granvin displayed three narrow clusters, with a broad cluster between the second and third narrow clusters. Stryn displayed a similar pattern in both 2017 and 2018, with two narrow clusters with broader clusters around them.

The first major peak occurred around day of year 125–130 in all rivers, corresponding to May 6th–May 11th, though Granvin had a smaller peak in migration around day of year 110 (April 21st). The second major peak was much more variable in its timing and occurred anywhere between 135 to 150 days into the year.

### Environmental correlates of migration

**Figure 3:**
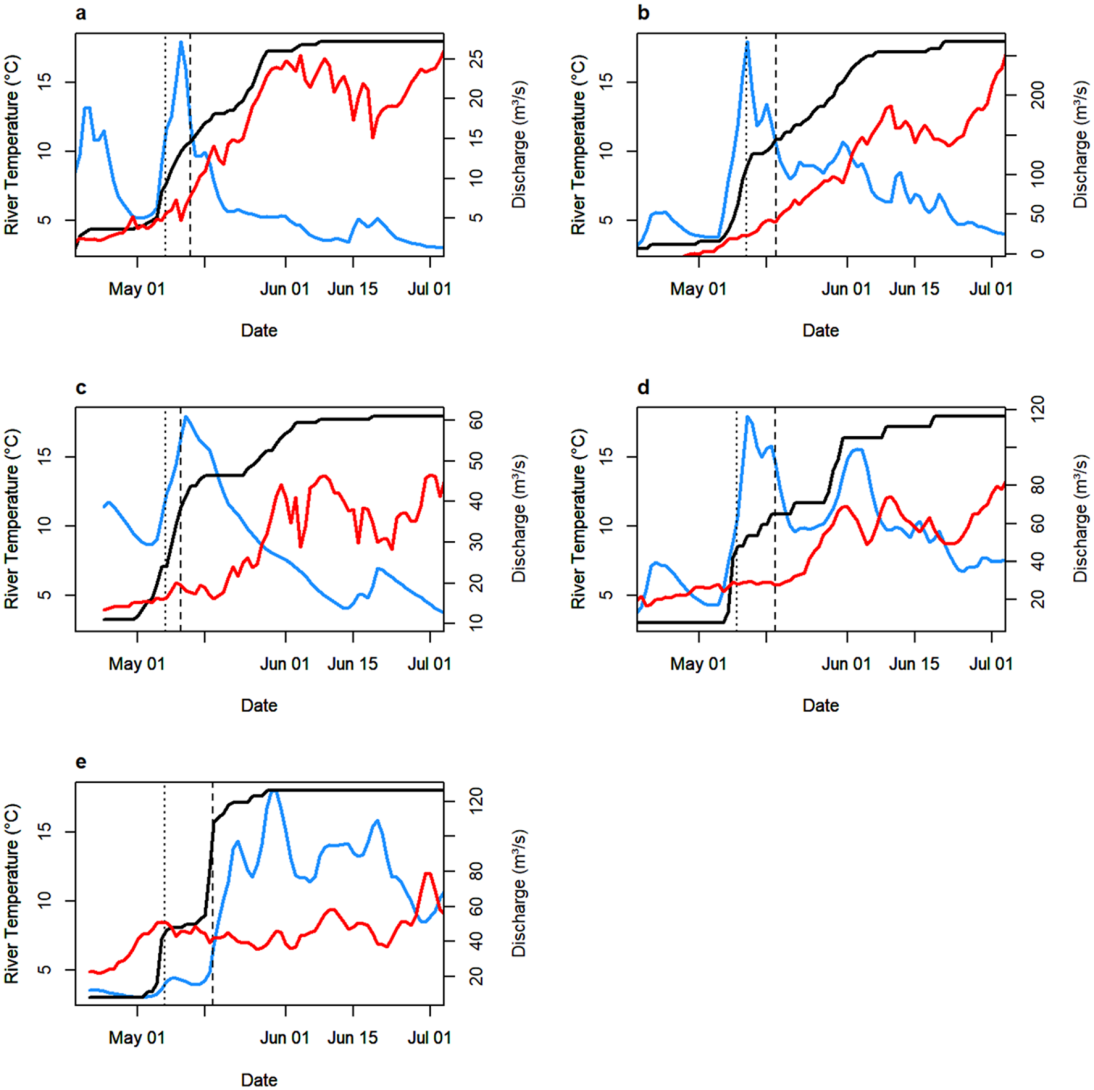
Mean daily water discharge (blue), mean daily temperature (red), and cumulative migration curve (black) through the migration period for (**a**) Granvin 2018, (**b**) Eio 2018, (**c**) Eid 2018, (**d**) Stryn 2018, and (**e**) Stryn 2017. 25% and 50% migration dates are shown as dotted and dashed lines, respectively.

The first major peak in migration seemed to coincide with a peak in discharge in all four rivers (Fig. 3).

Generalized linear mixed modelling fitted to fjord entry dates revealed that the most supported model included the effects of temperature, discharge, day-to-day change in discharge, and an interaction term between discharge and change in discharge. This model also included a random effect of river-year, such that the intercept was allowed to vary between river-years, but not slopes. Substantial collinearity (r_P_ = 0.803) between temperature and day of year necessitated fitting these variables in separate models. The model with the effect of temperature rather than day of year had the lower AIC (deltaAIC = 6.62).

**Figure 4:**
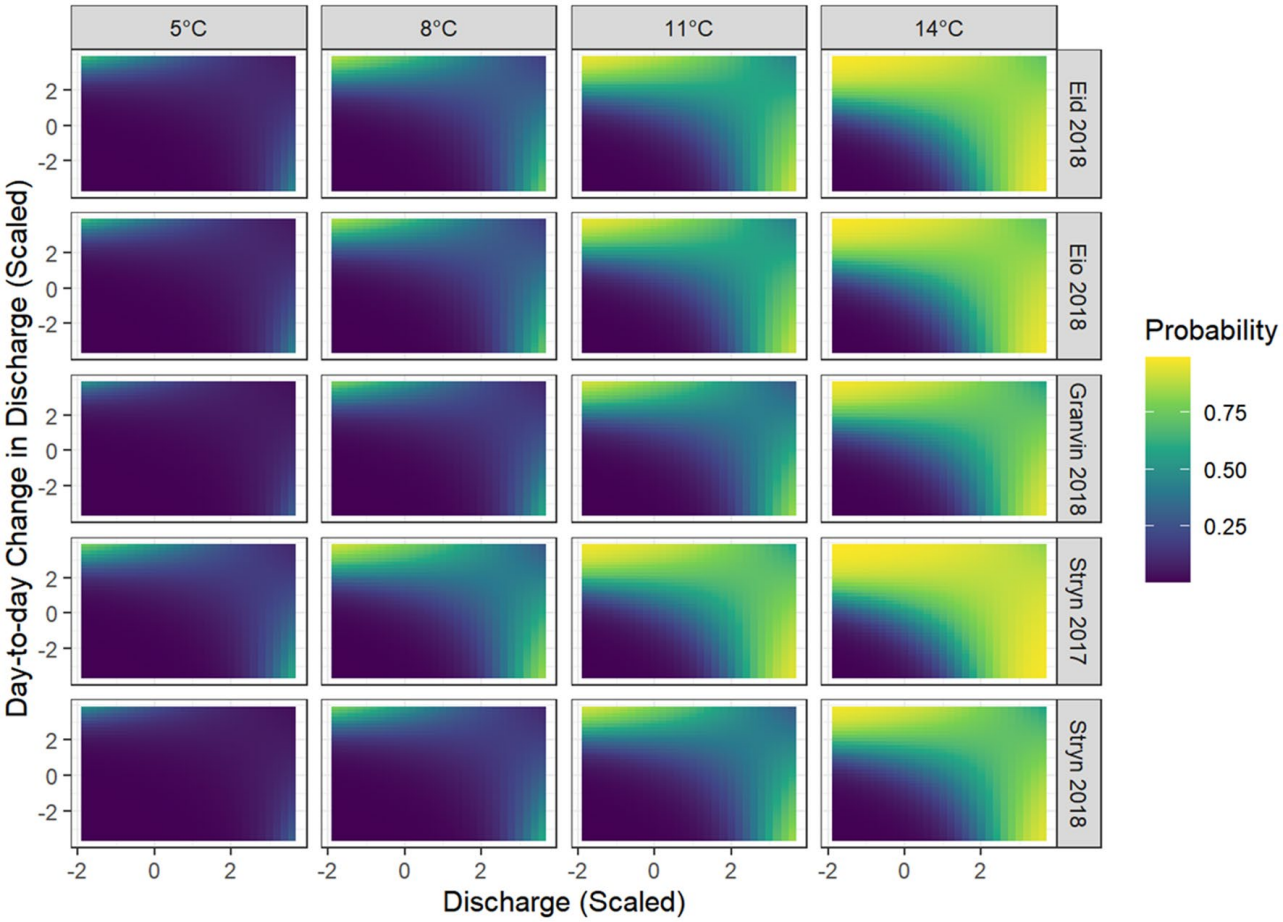
Migration probability predictions from best model of migration triggers. Yellow indicates a high probability of migration and blue indicates a low probability of migration given a set of river conditions.

The estimates/predictions of this model indicate that migration probability is initially only high when the discharge is low and the day-to-day change in discharge is positive. However, as the temperature increases through the season, the migration probability increases under all river conditions, but especially for cases where the discharge is high and the day-to-day change in discharge is negative (Fig. 4).

### Migration

**Figure 5:**
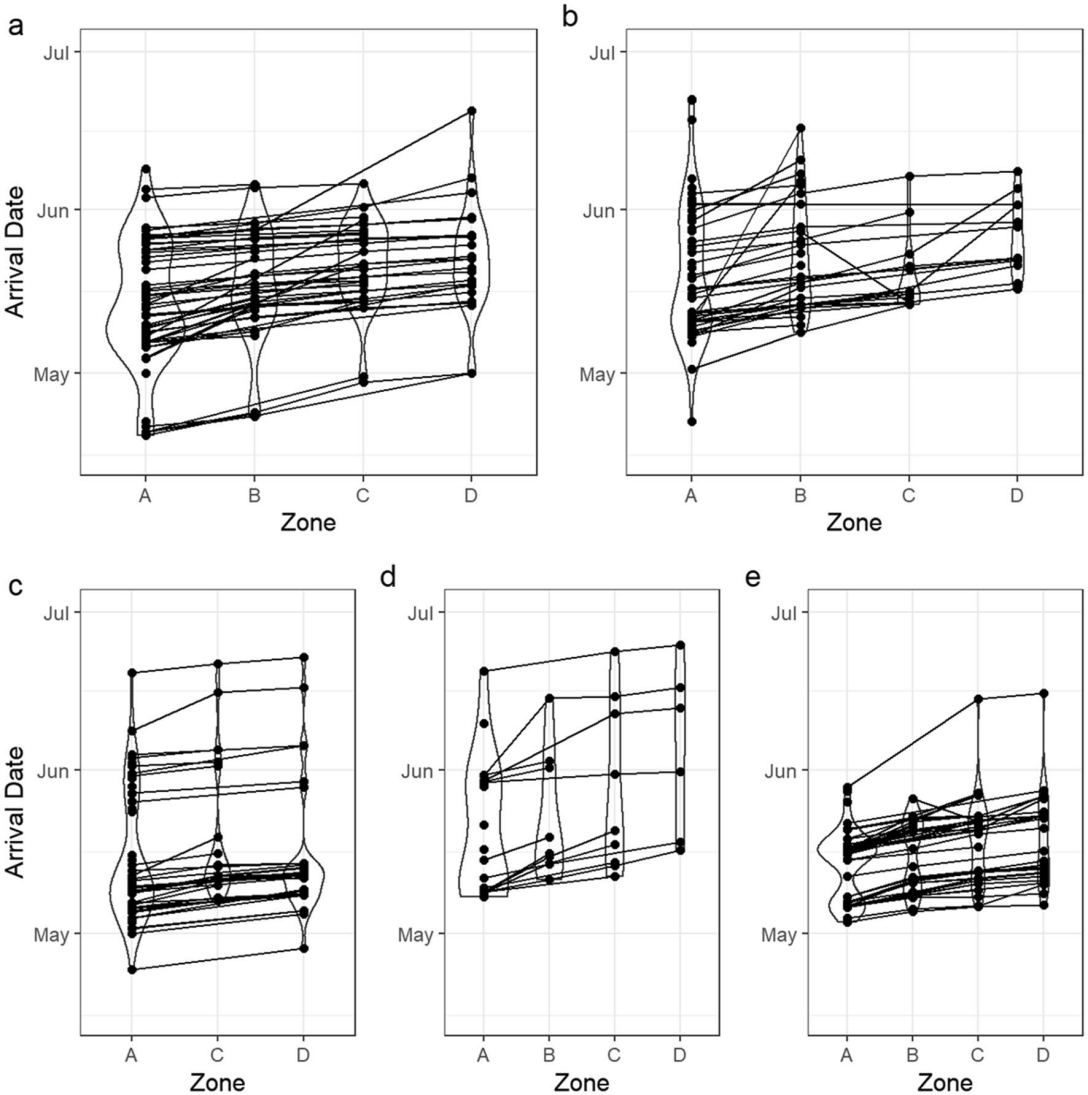
Arrival dates in each zone of the fjord for all migrators from (**a**) Granvin 2018, (**b**) Eio 2018, (**c**) Eid 2018, (**d**) Stryn 2018, and (**e**) Stryn 2017. Each line represents an individual fish.

The majority of smolts from all rivers moved through the fjord in a very directional manner (Fig. 5).

**Figure 6:**
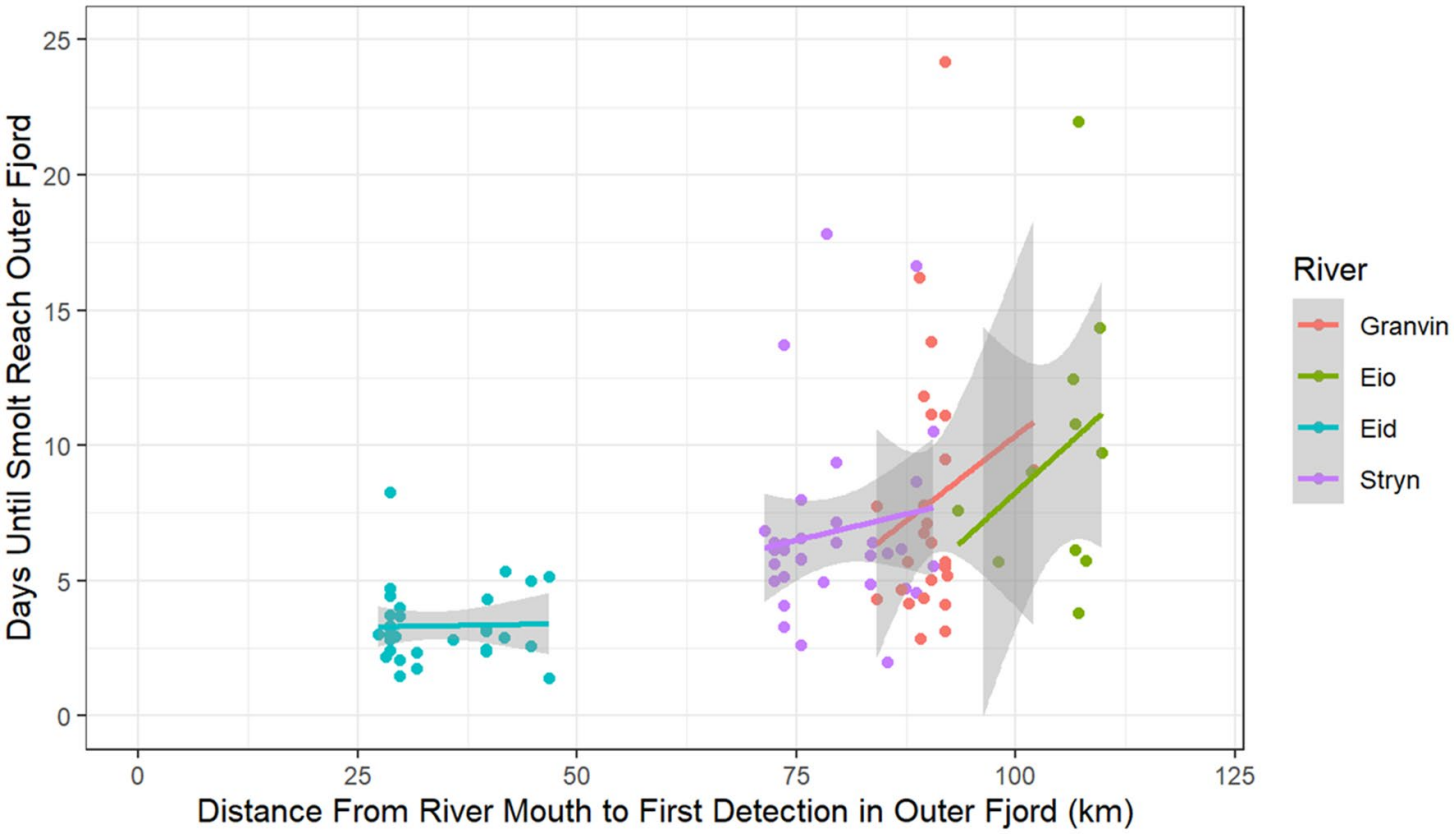
Travel time in days between the river mouth and the outer fjord as a function of the distance between the river mouth and the first receiver in the outer fjord at which each individual was detected, where the outer fjord is represented by zone D. Colored lines represent the results of river-specific linear regressions along with associated 95% confidence intervals.

However, there was substantial between-individual variation in the time spent to reach the outer fjord such that smolts originating from the same river diverged by up to 21 days to reach the outer fjord (Fig. 6). This between-individual variation in migration duration increased for populations with longer fjord migrations, such that the coefficient of variation increased from 0.40 in the smolts originating from the outermost river of Eid to 0.60 in smolts from the innermost river of Eio. Variation in the location of the first receiver that each smolt was detected at in zone D did not explain this variation (Fig. 6).

### Survival analysis

Overall, 38% of migrating smolts were detected successfully migrating to the outer fjord. However, due to imperfect detection rates in the receiver network, this value is an underestimate of total survival. The most supported model for detection probability allowed detection probability to vary among fjord zones. With data from Granvin and Eio, the most supported model for survival probability allowed survival probability to vary among zones and rivers, though models also including the effects of fjord entry date, sun position at fjord entry, and/or smolt length were not significantly worse (deltaAICc < 2). With only data from Eid, the best model included only the effect of smolt length, though the model that also allowed survival rates to differ between zones was not significantly worse (deltaAICc < 2). Using both years of data from Stryn, the best model had a constant probability of survival through the fjord, though models including the effects of length and/or zone were not significantly worse (deltaAICc < 2) (Supplementary Table S2).

Probability of detection in the first zone was at or near 1 in all models. This indicates that nearly all smolts were detected in or near the mouth of the river. Throughout the rest of both fjords, probability of detection varied from 0.42 to 0.82 (Supplementary Table S3).

**Figure 7:**
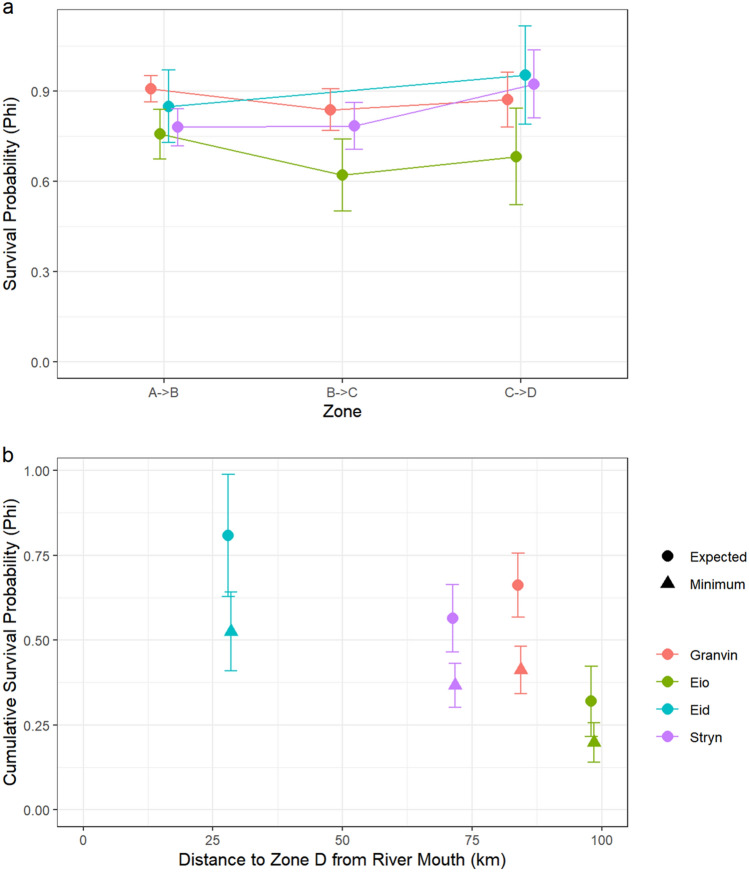
(**a**) Estimated probability of survival between each zone of the fjord for all rivers. Error bars represent standard error of estimates. Survival probabilities in the last transition are expected survival probabilities based on a best guess of the probability of detection equal to 0.65. (**b**) Minimum and expected cumulative survival probabilities for the entire fjord migration for each river as a function of the distance from the river mouth to the last zone. Error bars represent standard error of estimates.

Smolts originating from Eio consistently experienced lower survival rates throughout the fjord (Fig. 7a), but by assessing overlap of confidence intervals there were no significant differences between populations in any zone, nor were there significant differences between zones. However, cumulative survival rates through the fjord indicated that longer fjord migrations lead to lower cumulative fjord survival (Fig. 7b, Supplementary Table S4).

The proportion of individuals that were positively identified as having died within receiver range served as a check on survival rates as survival rates cannot exceed one minus this proportion. Only the expected survival rate for migrators from Eid came close to this proportion (Supplementary Table S4).

## Discussion

In all four study rivers, smolts began their migration between late April and early June with clear multimodal distributions across all river-years. Though the median migration time differed by up to 5 days, the timing of the first major cluster of migration differed only by 2 days among rivers in 2018 (Supplementary Table S1). Spring floods occurred at roughly the same time in all rivers in 2018, and modelling showed that both discharge and the relative change in discharge were important correlates of migration, while temperature seemed more important later in the season. Estimated fjord survival rates ranged between 32 and 81% among rivers, with no clear survival bottlenecks within the fjord.

We show here that migration timing in these populations seems to be primarily influenced by the timing of river spring floods, though temperature seems more important as the season progresses. The importance of water discharge and temperature in triggering migration has been observed many times elsewhere^3,22,24–26,49,50^. Here, the degree to which river conditions control the phenology of migration timing is the likely cause of the observed synchronization among rivers with similar hydrological properties. As all of the studied rivers are primarily fed by snowmelt throughout the migration period, the same regional weather systems can lead to a near simultaneous upswing in snowmelt and subsequent discharge in each river.

The effect of water discharge on the timing of migration leads to clear multimodality in the distribution of migration timing in these populations. Such multimodality in smolt migration timing has been observed in many other systems (e.g.^3,25,30,50–52^). However, the mechanisms behind this multimodality and its ecological consequences are rarely discussed (though see Freshwater et al.^53^). Therefore, it remains unclear how individual smolts decide which peak in river discharge to migrate in. Three non-mutually exclusive hypotheses could explain this.

First, smolts could differ in a set of environmental thresholds that must be crossed for migration to trigger. A threshold model has been proposed to explain the phenomenon of partial migration^54^, and a similar model could explain the variability we see here. Here, smolts would require one or more environmental variables to cross some threshold before they initiate their migration. This threshold could then differ among individuals due to genetic variation or phenotypic plasticity. Here, we see that in each river-year, the second peak in river discharge is both steeper and taller than the first (Fig. 3), leaving open the possibility that fish that migrated on the first peak had a lower discharge threshold.

Second, individuals that migrated later may not have completed their parr-smolt transformation until the second peak in river discharge. Physiological preparedness for seawater entry is a prerequisite for smolt migration^19,55,56^. However, the physiological smolt window, i.e. the window of time when the salmon are physiologically prepared for migration, and the environmental smolt window, i.e. the window of time in which salmon could be triggered to begin the migration, may or may not be fully overlapping periods of time. The timing of these windows seems to be determined by different environmental and/or endogenous cues, where the parr-smolt transformation is primarily determined by photoperiod and temperature^12,19,57,58^. Given that temperature and photoperiod regimens can vary widely between salmon rivers, there must be between-population variation in the way these regulate smoltification, which may explain documented between-population variance in migration timing^59^. However, within-population variation in the timing of the parr-smolt transformation has not been properly investigated^59^.

Third, the observed multimodality may be the product of an evolutionary compromise between a bet hedging strategy and a synchronization strategy. Both the physiological smolt window and the environmental smolt window are evolved traits that allow smolts to arrive at their feeding areas at the optimal time. However, there is an inherent difficulty in determining the optimal migration time given the conditions within the river, as the conditions that determine the optimality are distant in both space and time. If migration triggers are unreliable predictors of the optimal migration time, we would expect fish to hedge their bets through stochastic reaction norms^60^. Simultaneously, migrating synchronously can reduce a smolt’s individual risk of predation through a predator swamping effect^61^. Migrating in discrete batches may represent a compromise between these two evolutionary pressures. However, theoretical modelling would be necessary to evaluate whether such a compromise would be evolutionarily stable.

Additionally, common-garden experiments show that farmed and farmed-wild intraspecies hybrid smolts differ from wild smolts in their migration timing^30^, such that genetic introgression from domesticated salmon will likely increase within-population variation in migration timing. As all of the studied populations have experienced some degree of introgression^62^, this may explain some portion of the observed within-population variation. Further work is needed to establish whether within-population variation in the physiological smolt window exists and to what degree genetic variation can influence both the physiological smolt window and how smolts react to migration triggers.

Expected cumulative survival rates through the fjord ranged between 32 and 81%. Despite relatively high tag burdens, these results are in line with previous results showing survival through the early marine migration to range between 29 and 92% across a variety of rivers^13^. Populations situated further from the coast experienced lower survival rates through the fjord, likely explaining some portion of the decrease in the population density of salmon populations located farther from the coast observed by Vollset et al.^63^. Smolt length seemed to have a positive effect on fjord survival, likely in part due to lower tag burdens for larger fish. That said, the effects of tagging should diminish over time such that fish have likely adjusted by the time they migrate^64^, and similar tag burdens have been shown to have no effect on survival of juvenile salmon^65^. We observed that there was no statistically significant difference in tag burden between those that were detected as migrants and those that were not (t test, p = 0.1375). Therefore, we expect that the effect of smolt length on fjord survival is not solely due the effects of tag burden. Another likely mechanism for this relationship is that increased smolt length reduces the likelihood of predation by gape-limited predators^66,67^. As post-smolts are believed to be under the greatest risk of predation while they migrate^66^, it is not unexpected that a relationship between smolt length and survival would manifest itself in the fjord migration.

There was little support that migration timing had an effect on fjord survival as no candidate models including the effect of fjord entry date proved significantly better than other models, though nor were they significantly worse in the candidate models for smolts from Granvin and Eio. However, it is important to stress that the mortality estimated here likely comes before any salmon lice-induced mortality. Salmon post-smolts are unlikely to be infested by salmon lice until they reach the outer fjord where the salmon lice concentrations are highest^68^. Additionally, there will likely be a time lag between salmon lice infestation and any resulting mortality. As a result, smolts will likely be outside of our receiver network before any salmon lice-induced mortality can occur. This means that variation in migration timing will probably have a greater effect on return rates than fjord survival, especially in years where salmon lice densities are high^17^.

Similarly, there were no apparent benefits of synchronized migration as candidate models that included the effect of the maximum posterior probability of belonging to a narrow cluster proved to be significantly worse in all three datasets. However, benefits of synchronized migration should mostly manifest themselves during river migration where smolts are confined to relatively small spaces that can be exploited by opportunistic predators. Given that the observed estuarine residence times were negligible and that the length of the rivers are short, synchronized migration may not present a significant benefit for migrating smolts in these populations. Also, as the analysis only used smolts that could be positively identified as migrators, estimated mortality rates do not include mortality in the river. Further, there is some evidence that migrating salmon only school when migrating during the day^69^. As the majority of the smolts in this study began their migration during relative darkness (Supplementary Figures S2, S3), the predator swamping strategy may not be necessary.

There were no significant differences in survival rates between different sections of the fjord or between rivers. Interestingly, we observed that smolts originating from Eio consistently experienced lower mortality rates than smolts originating from Granvin in each zone of the fjord, despite that only zone A is different for these two populations. In other words, the 20 extra kilometers that post-smolts from Eio needed to traverse in zone A seemed to have a carryover effect into the other zones, leading to reduced survival throughout the fjord. This result could be partially explained by delays between the predation of a tagged fish and gastric expulsion of the tag leading to apparent smolt movement between zones^70^, but it could also indicate that exhaustion may be a cause of death.

Unsurprisingly, mean travel times to the outer fjord increased when populations were situated further from the outer fjord. However, within-population variation in travel times also increased such that fjord residency times for individuals from the innermost river varied by up to 3 weeks. Such large within-population differences in travel time through the fjord have been observed previously^71^ and it is worth investigating the underlying causes of this variation as it may lead to large differences in an individual’s risk of infestation by salmon lice. This effect likely plays some part in the observation that post-smolts caught in trawls of the outer fjord in the latter part of the season are primarily coming from the inner fjord^72^.

Migration times were consistent with previous research showing that salmon smolts from this area of Norway predominantly migrate in May^1,30^. However, median migration dates were on average 10.8 days earlier than those estimated previously by extrapolation from the median migration dates in neighboring rivers^36^. More years of data would be necessary to determine whether this difference is consistent.

In conclusion, we found that explicit consideration of the multimodality of migration timing improved our understanding of both within- and between-population variation of this trait. If the modes in migration timing can be easily predicted from readily available environmental data in other years, then it may be possible to provide live data to managers on when the majority of smolt are beginning their migration. Future research should focus efforts on understanding the within-population variation inherent to this modality and not only between-population variation, as understanding this will be paramount to understanding how populations will react to changing environmental conditions. Especially, an investigation of the degree to which genetic variation structures migration timing will be necessary to understand the capacity salmon have to evolve this trait.

## Supplementary information

Supplementary information.
