# Supplementary material for: Synchrony and multimodality in the timing of Atlantic salmon smolt migration in two Norwegian fjords
Source: Sci Rep. 2021 Mar 22;11:6504. doi: 10.1038/s41598-021-85941-9 (PMC7985142; doi:10.1038/s41598-021-85941-9)
Supplement: Supplementary file 1 — Supplementary information. [file 41598_2021_85941_MOESM1_ESM.pdf]

Synchrony and multimodality in the timing of Atlantic salmon smolt migration in two Norwegian fjords

Helge B. Bjerck, Nord University, Faculty of Biosciences & Aquaculture, helge.b.bjerck@nord.no*

Henning A. Urke, INAQ AS, henning.urke@inaq.no

Thrond O. Haugen, Norwegian University of Life Sciences (NMBU), Faculty of Environmental Sciences & Natural Resource Management, thrond.haugen@nmbu.no

Jo Arve Alfredsen, Norwegian University of Science & Technology (NTNU), Faculty of Information Technology & Electrical Engineering, jo.arve.alfredsen@ntnu.no

John Birger Ulvund, Nord University, Faculty of Biosciences & Aquaculture, john.b.ulvund@nord.no

Torstein Kristensen, Nord University, Faculty of Biosciences & Aquaculture, torstein.kristensen@nord.no

Supplementary Information

Gaussian Clustering of Fjord Entry Dates

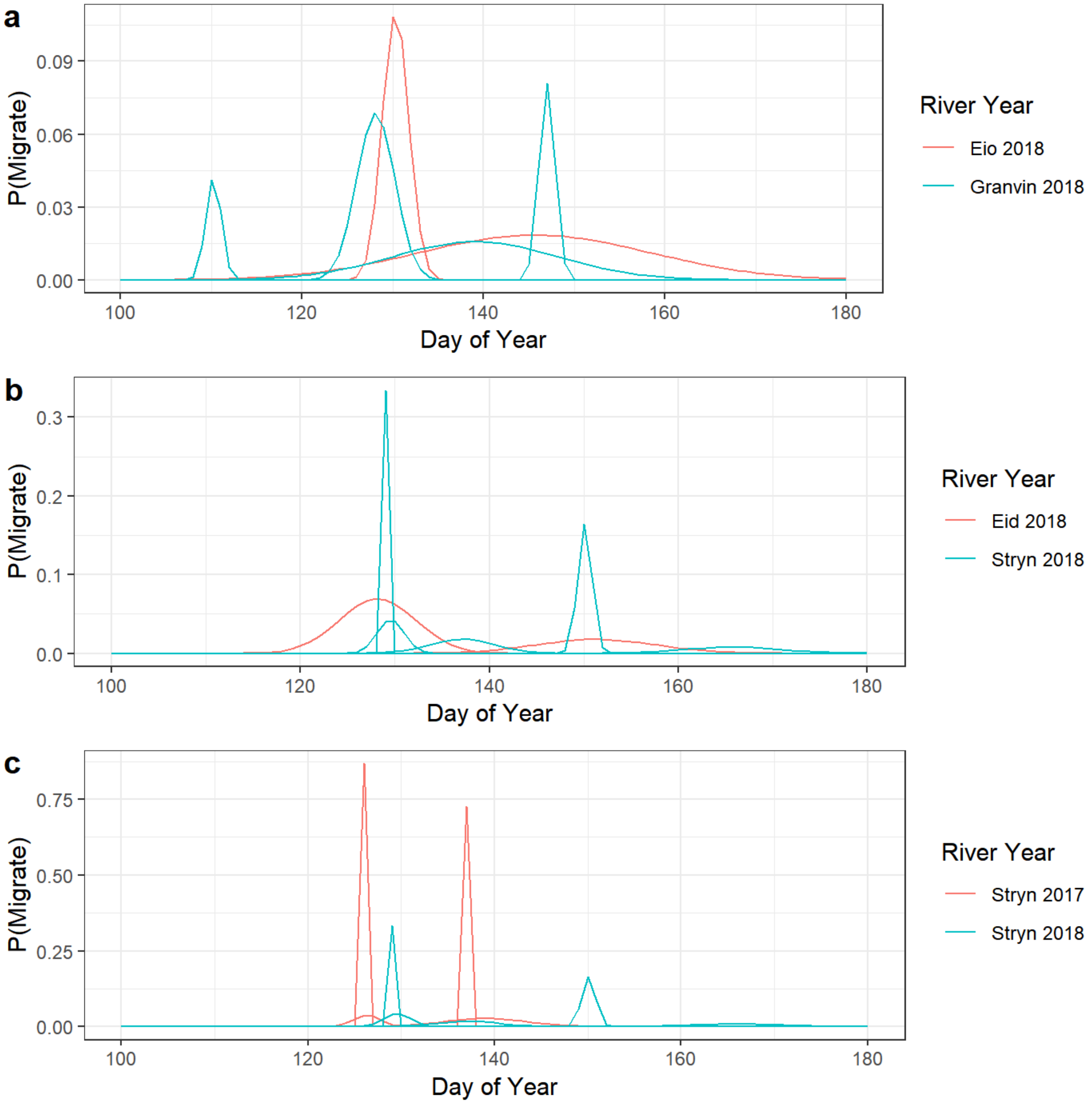

Figure S1: Normal distributions resulting from Gaussian clustering of fjord entry dates for both rivers in (a) Hardangerfjord, both rivers in (b) Nordfjord, and for both years of data from (c) Stryn.

Table S1: Summary statistics of all clusters generated by gaussian clustering of fjord entry dates for each river year

River Year	Cluster #	# of ID's Assigned to Cluster	Proportion of Migrators Assigned to Cluster	Mean (Day of Year)	St. Dev.
Eid 2018	1	39	0.66	128.3	3.24
	2	20	0.34	144.9	12.54
Eio 2018	1	23	0.40	130.3	1.45
	2	34	0.60	145.6	12.82
Granvin 2018	1	6	0.09	110.3	0.84
	2	24	0.36	128.1	2.11
	3	24	0.36	139.1	9.20
	4	12	0.18	147.0	0.90
Stryn 2017	1	14	0.20	126.1	0.07
	2	10	0.14	126.3	1.45
	3	25	0.35	137.0	0.19
	4	22	0.31	138.9	4.53
Stryn 2018	1	5	0.26	128.9	0.06
	2	3	0.16	129.5	1.44
	3	3	0.16	137.1	3.49
	4	6	0.32	150.1	0.76
	5	2	0.11	165.8	5.15

Time-of-day of Migration Initiation

Within-day timing of the initiation of migration was determined with the aid of the R package suncalc[74]. The function getSunlightPosition in this package provides the position of the sun with respect to the horizon through time, given a latitude and longitude. Owing to the high latitude of the study system, the angle at which the sun moves with respect to the horizon is very acute such that twilight represents a significant portion of the day. As a result, considering only sunrise/sunset when investigating diel patterns might inaccurately represent when night and day begin. Two commonly used breakpoints are nautical and civil twilight. Nautical twilight is the period of time where the sun is between 6 and 12 degrees below the horizon, such that light is visible on the horizon but there is not sufficient light for human eyes to distinguish objects. Civil twilight is the period of time where the sun is at most 6 degrees below the horizon such that human vision is sufficient to discern objects.

Smolt seemed to initiate their migration during nighttime when this option was available (Figure S2). However, after day of year 130 (May 10th) at this latitude, there is always some level of light visible on the horizon. As a result, migration under true darkness becomes impossible. Around day of year 140 (May 20th), the proportion of the day that is between dawn and dusk is roughly in concordance with the proportion of migrators that migrate in this period (Figure S3).

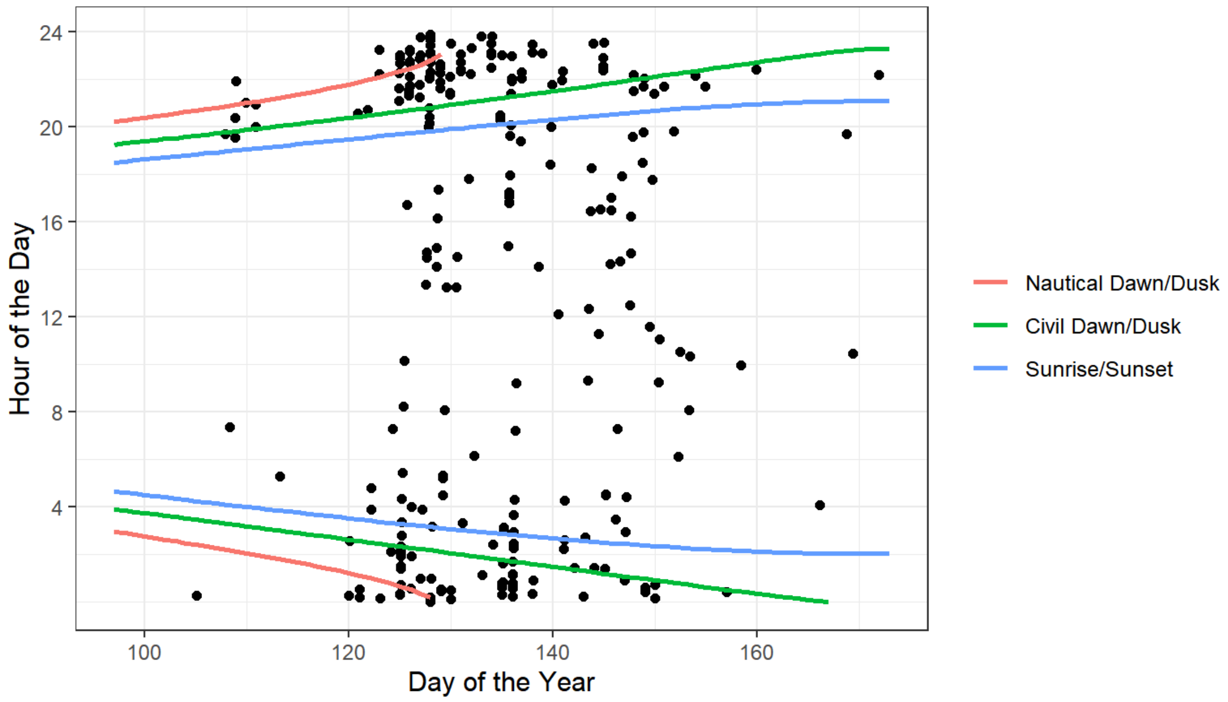

Figure S2: Time of day at fjord entry with respect to day of year for all migrators from all populations. Red, green, and blue lines represent the start/end times of nautical dawn/dusk, civil dawn/dusk, and sunrise/sunset, respectively.

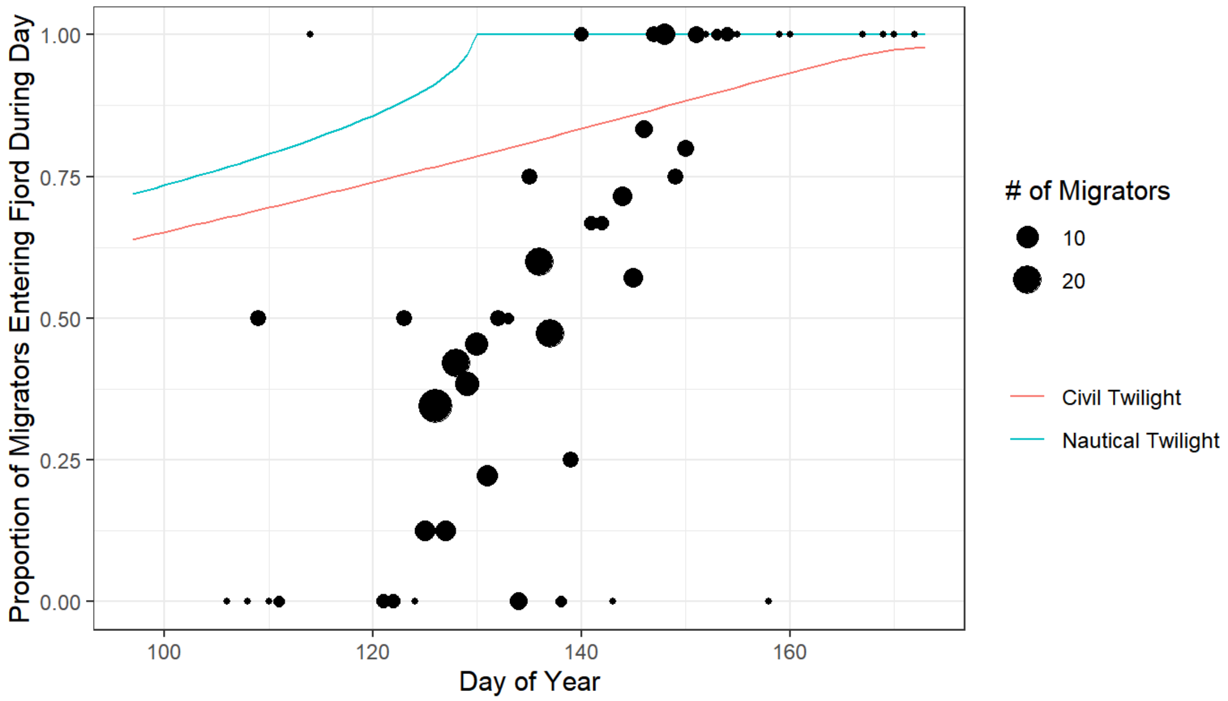

Figure S3: Proportion of migrators from all rivers that enter the fjord between civil dawn and dusk for each day. Size of points indicate the number of smolts that migrate on that day. Red and blue lines indicate the proportion of the day with at least civil and nautical twilight levels of light, respectively.

Survival Analysis

Table S2: Model selection results of Cormack-Jolly-Seber modelling. Population indicates in which population(s) these models were tested and Model Structure shows the tested model for both probability of survival (phi) and probability of detection (p). The number of parameters indicates how many parameters needed to be estimated in said model, while the AICc is the Akaike information criterion with a correction for small sample size and DeltaAICc shows the difference in AICc values between this model and the best model. In the model structure, abbreviations are used for some variables: FED is Fjord Entry Day, SP is the Position of the Sun with respect to the horizon, MPN is the Maximum Probability of an individual belonging to a Narrow cluster.

Population(s)	Model Structure	# of Parameters	AICc	DeltaAICc
Granvin & Eio	Phi ~ zone + river, p ~ zone	8	500.1387	0.00
	Phi ~ zone + river + FED, p ~ zone	9	500.3887	0.25
	Phi ~zone + river + length, p ~ zone	9	500.7010	0.56
	Phi ~ zone + river + FED + length, p ~ zone	10	501.2153	1.08
	Phi ~ zone + river + SP, p ~ zone	9	502.0414	1.90
	Phi ~ zone + MPN + river, p ~ zone	9	502.2413	2.10
	Phi ~ zone, p ~ zone	7	505.3613	5.22
	Phi ~ river, p ~ zone	5	510.8084	10.67
	Phi ~ 1, p ~ zone	4	582.7008	82.56
	Phi ~ 1, p ~ zone + river	5	583.4416	83.30
Eid	Phi ~ length, p ~ zone	4	144.2799	0.00
	Phi ~ zone + length, p ~ zone	6	145.2844	1.00
	Phi ~ zone + FED + length, p ~ zone	7	147.4451	3.17
	Phi ~ length + SP, p ~ zone	6	148.5975	4.32
	Phi ~ 1, p ~ zone	3	165.0434	20.76
	Phi ~ zone + FED, p ~ zone	6	166.8358	22.56
	Phi ~ zone + MPN, p ~ zone	6	166.8849	22.60
	Phi ~ zone, p ~ zone	5	167.7581	23.48
Stryn	Phi ~ 1, p ~ zone	4	349.1106	0.00
	Phi ~ length, p ~ zone	5	349.4030	0.29
	Phi ~ zone + length, p ~ zone	8	350.6499	1.54

	Phi ~ zone + FED + length, p ~ zone	9	352.2337	3.12
	Phi ~ zone + FED, p ~ zone	8	352.2978	3.19
	Phi ~ zone + SP, p ~ zone	8	352.3785	3.27
	Phi ~ zone + MPN, p ~ zone	8	352.7090	3.60
	Phi ~ zone + year, p ~ zone	8	352.7169	3.61
	Phi ~ 1, p ~ 1	2	390.0186	40.91
	Phi ~ 1, p ~ zone + year	5	396.7523	47.64

Table S3: Estimated probability that a smolt is detected in each zone for each dataset, with standard errors

Zone	Hardanger	Eid	Stryn
A	0.98±0.011	1±0	1±0
B	0.65±0.060	NA	0.63±0.068
C	0.61±0.075	0.42±0.089	0.82±0.067

Table S4: Table of expected, minimum, and observed survival rates for each river, along with standard errors of estimates

River	Expected	Minimum	1-Observed Mortality
Granvin	0.662±0.094	0.412±0.07	0.847
Eio	0.32±0.104	0.198±0.058	0.930
Eid	0.808±0.18	0.525±0.117	0.955
Stryn	0.564±0.099	0.367±0.064	0.833

References

1. Thieurmel, B. & Elmarhraoui, A. *suncalc: Compute Sun Position, Sunlight Phases, Moon Position and Lunar Phase*. (2019).
